# EFFECT OF UNMANNED AERIAL VEHICLE MISSION DIFFICULTY LEVEL ON PILOT'S AUTONOMIC NERVOUS SYSTEM

**DOI:** 10.13075/ijomeh.1896.02630

**Published:** 2025

**Authors:** Przemysław Wojciechowski, Konrad Wojtowicz, Jan Błaszczyk

**Affiliations:** 1 Military University Of Technology, Faculty of Mechatronics, Armament and Aerospace, Warsaw, Poland; 2 Calisia University, Faculty of Health Sciences, Kalisz, Poland

**Keywords:** pilot, ECG, flight simulator, autonomic nervous system, UAV, PZT

## Abstract

**Objectives:**

The aim of this article is to investigate the psychophysiological responses of unmanned aerial vehicle (UAV) pilots during flight simulations with high mission complexity. In particular, it focuses on the responses of the sympathetic and parasympathetic parts of the autonomic nervous system.

**Material and Methods:**

Fourteen pilots aged 26–31 years took part in the study. The research was conducted using a UAV flight simulator. During the test, data was collected from electrocardiogram sensor and piezoelectric (lead zirconate titanate – PZT) respiration sensor as well as the pilot's mission performance was assessed. In addition, the test subjects were subjected to a simple reaction speed test after the completed exercise.

**Results:**

For missions classified as having low difficulty, the mean RR interval (meanRR) was 1004.03 milliseconds, with a standard deviation (SD) = 18.5 ms. This corresponds to an mean heart rate of about 59.8 bpm (SD = 1.1), which is 16.9% longer than the RR intervals observed during high difficulty missions (meanRR±SD 859±59.75 ms). The values of respiratory rate per minute for the different levels of difficulty were M±SD 17.3±0.87 for low, 18.1±1.04 for medium, and 18.8±0.41 for high mission difficulty.

**Conclusions:**

A correlation between the effects of an unmanned aircraft flight simulator and the pilot's body was proven. By means of tests using electrocardiogram, PZT and reaction time measurements, it was proven that the flight simulator directly induces stressful stimuli that affect the subject's body. By analyzing the individual results, it was also proven that the sympathetic part of the nervous system is activated as the level of mission difficulty increases.

## Highlights

- Flight simulator directly induces stressful stimuli that affect the subject's body.
- Activation of the sympathetic nervous system increases proportionally with rising mission difficulty.
- Visual-motor reaction time decreases as mission difficulty increases.

## INTRODUCTION

The development and popularization of unmanned aerial vehicles (UAVs) have revolutionized many fields. Both military missions and civilian operations are carried out with their help. With the increasing number of UAVs and the complexity of the missions performed by pilots, the need to understand the psychophysiological reactions in the pilots' bodies has been recognized. Understanding the aspects involved appears to be key to optimizing the performance and safety of the flights performed. This article focuses on the responses of the sympathetic and parasympathetic parts of the autonomic nervous system to the impact of a flight simulation of an unmanned aircraft with a high level of mission complexity [1–4].

The research is important because of its potential implications for drone pilot performance and accident prevention in general. Activating the sympathetic or parasympathetic nervous system is important for stress response, decision-making, and cognitive function. Understanding these responses regarding UAV piloting can provide clues to the physiological factors contributing to drone accidents and help develop accident prevention strategies.

Most research focuses on analyzing pilots' physiological responses during manned flight. Research targeting unmanned aerial vehicle pilots needs more attention with the increased frequency of UAVs use and future co-functioning with manned aviation.

The extant literature contains a paucity of research specifically addressing UAVs pilots, and, in particular, the response of their autonomic nervous systems to simulation-generated stimuli. A number of studies have been conducted on driving simulators [5–7], however, in this instance, the nature of the simulation is distinct. The simulator sessions, which are characterized by their monotony, do not accurately reflect the exercises that involve the use of simulators to pilot unmanned racer aircraft with first person view (FPV) imaging. As indicated in the literature, references to the psychophysical state of pilots during flight simulator training principally pertain to pilots of manned aircraft [8,9]. A plethora of studies have been conducted on the effects of flight simulators on various physiological parameters, including heart rate (HR), heart rate variability (HRV), respiration, and electroencephalogram (EEG). However, the majority of these studies have concentrated on the changes in these parameters in response to the workload levels of pilots [10,11], rather than on the difficulty level of the mission itself [8].

This study aims to fill these gaps in the literature. The authors assessed and compared the sympathetic and parasympathetic nervous system responses of drone pilots flying missions of varying difficulty levels. Through this research, the authors want to highlight the psychophysiological challenges that drone pilots face in their daily work.

### The autonomic nervous system

The autonomic nervous system is divided into the sympathetic and parasympathetic systems. These systems occur both at rest and during stressful situations. Their reactions are reciprocal; when one is activated, the other becomes quieter [12,13].

#### Mechanisms of action of the sympathetic nervous system

The sympathetic nervous system is responsible for the body's responses in stressful and threatening situations. It works on a fight-or-flight basis and influences many physiological functions, including breathing and the cardiovascular system [14–16]:

– neurotransmitters
  - norepinephrine is the sympathetic nervous system's primary neurotransmitter, released from nerve endings, and affects various adrenergic receptors in target tissues,
  - adrenaline is released from the adrenal medulla into the bloodstream, enhancing norepinephrine's action, adrenergic receptors,
  - α-adrenergic receptors are responsible for vasoconstriction, leading to increased blood pressure,
  - β-adrenergic receptors have different functions depending on the type β_1_-adrenergic receptors (β_1_-AR): increases heart rate (chronotropic), the strength of heart contractions (inotropic), and conduction of impulses (dromotropic), β_2_-adrenergic receptors (β_2_-AR) causes bronchodilation (bronchodilation) and smooth vascular muscle relaxation (in skeletal muscle areas),
– changes in respiratory parameters
  - bronchodilation: the effect on β_2_-AR causes bronchial smooth muscle relaxation, leading to increased airflow through the airways,
  - increase in respiratory rate: an increase in sympathetic activity increases respiratory rate, allowing faster delivery of oxygen to the tissues and removing carbon dioxide,
– changes in cardiovascular parameters
  - increase in heart rate: norepinephrine acting on β_1_-AR in the heart leads to accelerated heart rate (tachycardia),
  - increased strength of heart contractions: the action of norepinephrine on β_1_-AR increases the strength of heart contractions, leading to an increase in stroke volume and cardiac minute volume,
  - vasoconstriction: norepinephrine acting on α-adrenergic receptors causes vasoconstriction, increasing peripheral resistance and blood pressure,
  - redirection of blood flow: the vasoconstriction of blood vessels in areas that are not relevant at the moment (e.g., skin, digestive system) and the vasodilation of vessels in key areas (e.g., skeletal muscles, heart) enables the body to use its energy resources in stressful situations optimally.

#### Mechanisms of action of the parasympathetic nervous system

The parasympathetic nervous system acts in opposition to the sympathetic nervous system. It is responsible for restoring and maintaining the body's homeostasis and the “rest and digest” response [14–16]

– neurotransmitters
  - acetylcholine is the parasympathetic neurotransmitter released from nerve endings and affects cholinergic receptors in target tissues,
– cholinergic receptors
  - muscarinic receptors are responsible for most of the effects of acetylcholine in the parasympathetic system,
  - nicotinic receptors can be found mainly in autonomic ganglia and skeletal muscles,
  - changes in respiratory parameters,
  - bronchospasm is an action on muscarinic receptors that causes bronchial smooth muscle contraction, decreasing airflow through the airways,
  - decrease in respiratory rate: a decrease in sympathetic nervous system activity and an increase in parasympathetic activity results in a decrease in respiratory rate, contributing to energy conservation and recovery,
– changes in cardiovascular parameters
  - decrease in heart rate: acetylcholine acting on muscarinic receptors in the heart leads to a slowing heart rate (bradycardia),
  - decrease in the strength of heart contractions: the action of acetylcholine on muscarinic receptors causes a decrease in the strength of heart contractions, leading to a decrease in ejection volume and cardiac minute volume,
  - vasodilation: acetylcholine acting on muscarinic receptors in blood vessels causes smooth muscle relaxation, leading to vasodilation and decreased peripheral resistance,
  - increased blood flow to the digestive organs: an increase in parasympathetic activity redirects blood flow to the digestive system, promoting digestion and nutrient absorption.

## MATERIAL AND METHODS

### Experimental participants

The study was conducted on a group of 14 male UAVs pilots, individuals with varying piloting skills. However, in order to create the study group, an effort was made to select individuals with similar experience of flying UAVs. Furthermore, it was observed that the pilots primarily flew UAVs of the same type. The mean age of the subjects was 27.1 years old, with the youngest pilot being 26 years old and the oldest 31 years old. Each subject reported no psycho-physical complaints at the time of testing that could potentially affect test results. The test subjects never experienced symptoms of motion sickness. The subjects were rested on the day of testing, had not taken any medication or alcohol, and had not exercised before testing (so as not to artificially affect cortisol levels). In addition, all had previous experience flying unmanned quadcopter aircraft using the FPV system and the Liftoff simulator.

The execution of the study was preceded by the issuance of consent to conduct the study by the Bioethics Committee at the Military Medical Chamber in Warsaw (Resolution No. 20/23 of the Bioethics Committee at the Military Medical Chamber in Warsaw dated July 14, 2023 on issuing an opinion on the medical experiment project).

### Software and apparatus used in the study

The study used Liftoff, an existing UAV racing drone simulator. The system uses advanced drone models validated through empirical testing and computational fluid dynamics (CFD) simulations. In addition, the simulated drones are configurable, and their components are designed according to factual specifications, which makes the simulated flights highly realistic [17,18].

The drone simulator was run on a Dell Precision laptop (Dell, Round Rock, TX, USA) (13th generation Intel(R) Core(TM) i9-13950HX 2.20 GHz [Intel, Santa Clara, CA, USA]; 64 GB RAM; NVIDIA RTX 3500 [NVIDIA Corporation, Santa Clara, CA, USA]) with Windows 11 Pro (Microsoft, Redmond, WA, USA) operating system.

**Figure 1:**
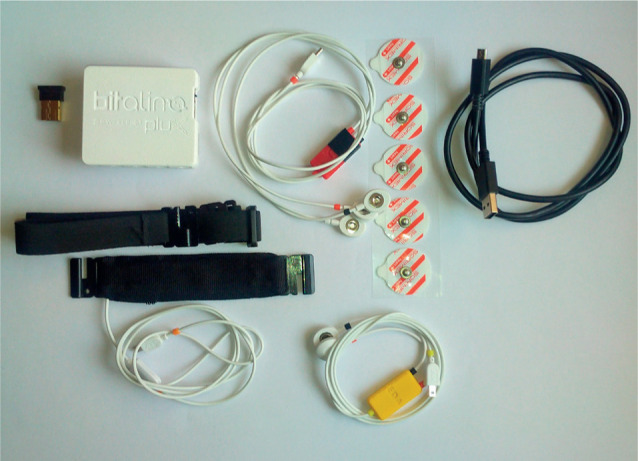
BITalino PsychoBIT module used in the research on group of 14 male unmanned aerial vehicles pilots, Military University of Technology, Warsaw, Poland, April 2025

The BITalino PsychoBIT kit (BITalino, Lisbon, Portugal) was used for biomedical data acquisition (Figure 1). The tests focused on measuring heart rate variability and the pilot's respiratory signals.

Photo: Przemysław Wojciechowski

Each time after the flight, the pilot performed an additional test to check reaction time (visual-motor RT). The test was conducted using a program available on the Arealme website [19].

The BITalino PsychoBIT kit consists of a main chip, the BITalinoCore BT, which allows Bluetooth communication with a computer to collect measurements. The BITalino-Core BT has six analog channels that allow the connection of sensors such as electrocardiogram (ECG) sensor, electrodermal activity (EDA) sensor, piezoelectric (lead zirconate titanate – PZT) respiration sensor, pulse sensor, and others [20].

**Figure 2:**
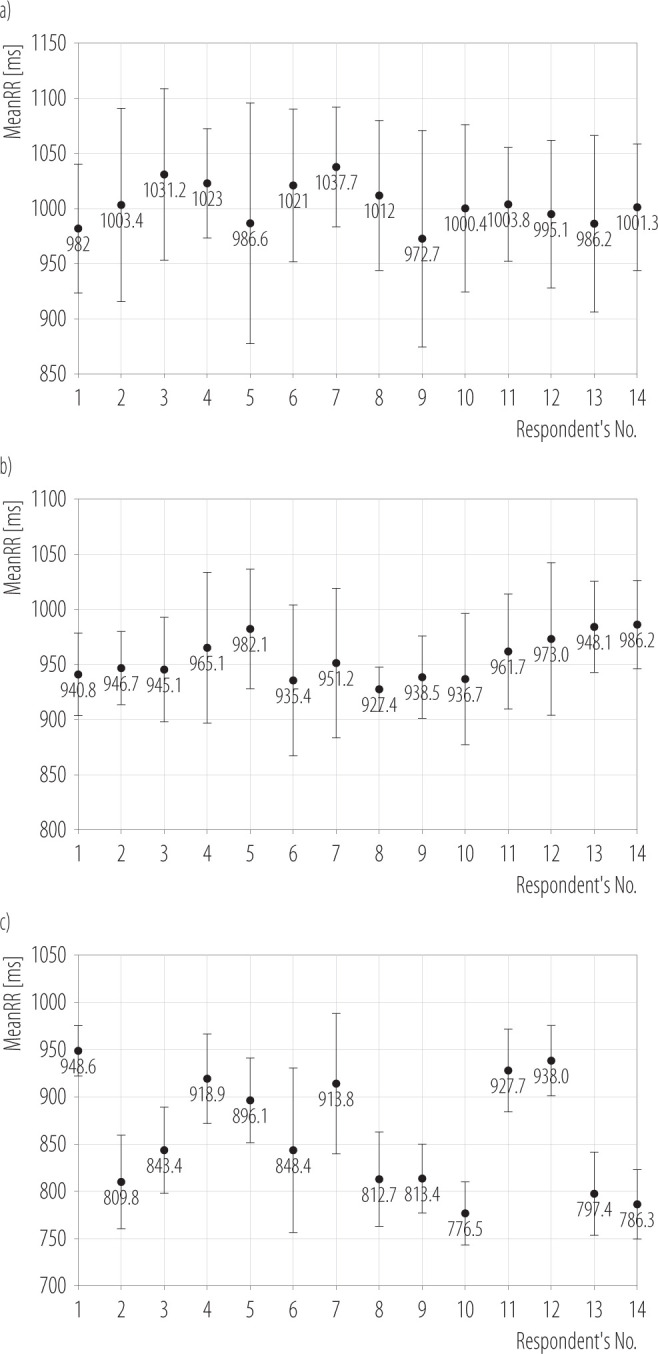
The mean RR interval (meanRR) of individual respondents at a) low, b) medium, and c) high level of mission difficulty, in the research on group of 14 male unmanned aerial vehicles pilots (mean age 27.1 years), Military University of Technology, Warsaw, Poland, April 2025

Electrocardiogram and PZT sensors were used in the study. Figure 2 shows an example of electrode placement and the BITalino module.

The basic technical parameters of the ECG sensor are [21,22]:

– gain 1100 – determines how much the input signal will be amplified before processing,
– range ±1.5 mV – the range of voltages the sensor can record, with a supply voltage of 3.3 V,
– frequency response 0.5–40 Hz – the minimum and maximum frequency of the signals the sensor can record,
– power consumption ~0.17 mA – the current consumed by the sensor during operation,
– input voltage range 2.0–3.5 V – the supply voltage at which the sensor operates correctly,
– input impedance 7.5 GΩ – the input resistance of the sensor,
– interference suppression index 86 dB – the system's ability to attenuate the same noise on both signal inputs.

### Respiratory sensor

The PZT sensor can determine whether the subject inhales or exhales. It is housed in a flexible band that can be adjusted to fit the user's chest. Its electrical output signal in proportion to the mechanical stress acting under the rising or falling of the human chest.

The parameters of the PZT sensor are:

– reinforcement 1,
– range ±1.5 V,
– frequency response 0.59–0.9 Hz,
– power consumption 35 µA [23–25].

### Research procedure

Three maps with different levels of difficulty were selected for the survey. The pilots unanimously labeled each route profile according to easy, medium, and difficult categories. Pilot had to pass all the checkpoints as quickly as possible during each test. Missing any point prevented completion of the test. Each time the pilot performed a mission, he was connected to an ECG and a PZT sensor. Data from the ECG and PZT sensors were recorded during each flight and entered into computer memory. After completing the task, the participant performed a visual-motor RT test 5 times. The ECG and PZT were not measured during the test. This was due to the dynamic nature of the visualmotor RT test, which could have distorted the results. It was followed by a 10-minute rest, after which the test subject was asked to repeat the test at a given level of route difficulty. Nevertheless, in order to mitigate the potential impact of pilot fatigue on their ability to pilot the aircraft, it was imperative to discontinue the tests if the pilot exhibited signs of fatigue or self-reported fatigue. The subsequent day's tests were scheduled for the afternoon, ensuring a consistent and uninterrupted evaluation process. Each pilot performed 10 flights on each of the 3 routes, which allowed data from 420 flights to be analyzed.

Parameters directly reflecting the heart rhythm were selected to interpret the ECG results. The analysis focused on measurements taken in both the frequency and time domains. Table 1 shows the parameters analyzed with an explanation of their significance.

**Table 1:** List of heart rate variability parameters and their significance [26–29]

Parameter	Meaning
Heart rate variability	
time domain	
minRR	minimum temporal interval between consecutive vertices R [ms]
maxRR	maximum temporal interval between consecutive vertices R [ms]
meanRR	mean of the temporal intervals R to R [ms]
SDRR	standard deviation of the mean of the temporal intervals R to R [ms]
meanHR	mean heart rate [bpm]
RR20	number of differences between consecutive R to R intervals >20 ms
pRR20	percentage of differences >20 ms [%]
RR50	number of differences between consecutive N to N intervals >50 ms
pRR50	percentage of differences >20 ms [%]
frequency domain	
LF power	value of the spectral power density for the low-frequency band to the total spectral power [%]
HF power	value of the spectral power density for the high-frequency range to the total spectral power [%]
TOTAL power	total spectral power from the distribution [ms^2^/Hz]
LF/HF	ratio of the spectral power value in the low-frequency range to the spectral power in the high-frequency range
Respiratory	
RESP FREQ	respiratory rate based on the entire sensor signal [breaths/min]
FREQ	frequency for the maximum value from the power density distribution of the signal spectrum

Parameters directly reflecting the respiratory system were selected to interpret the PZT results. Parameters in the frequency domain determining respiratory and frequency from the spectral power density distribution were extracted as the most authoritative. Table 1 shows the parameters analyzed with an explanation of their significance.

## RESULTS

First, the results are presented by the mission difficulty level. The following section presents a summary of the results and how they compare to each other.

A tabular summary of the measurements' results was made for each difficulty level. Each pilot is represented by one row, showing the arithmetic average of the results obtained in the 10 measurement sessions performed at each mission difficulty level.

### Low level of mission difficulty

The mean RR value (meanRR) (Table 2, Figure 2a) from all measurements was M±SD 1004.1±71.7 ms. It corresponds to almost 60 bpm. The mean value of the spectral power density for the high-frequency (HF) range to the total spectral power of 3095.6% and the mean ratio of the spectral power values in the low-frequency (LF) range to the spectral power in the HF range of 0.26 indicate parasympathetic nervous system activity.

**Table 2:** Summary of parameters obtained with electrocardiogram and piezoelectric respiration sensors on group of 14 male unmanned aerial vehicles pilots, Military University of Technology, Warsaw, Poland, April 2025

Mission difficulty	RR [ms]				rMSSD [ms]	meanHR [bpm]	RR20 [n]	pRR20 [%]	RR50 [n]	pRR50 [%]	Power				RESP FREQ [breaths/min]	FREQ
	minRR	maxRR	meanRR	SDRR							LF [%]	HF [%]	total [ms^2^/Hz]	LF/HF		
Low difficulty level																
1	760	1110	982	58.1	63.8	61.1	31	41.9	9	12.2	476.2	1337.4	1967	0.4	17.65	0.29
2	530	1100	1003.4	87.8	116.5	59.8	42	50.6	14	16.9	1605.2	3707	5985.1	0.4	14.81	0.25
3	530	1130	1031.2	78.3	113.7	58.2	54	43.5	13	10.5	647.6	2236.7	3246.3	0.3	16.59	0.28
4	690	1110	1023	49.5	70.1	58.7	27	44.3	4	6.6	729.2	2754.9	3588.2	0.3	16.88	0.28
5	510	1140	986.6	108.9	143.3	60.8	58	54.2	20	18.7	262.7	1484.5	1872	0.2	17.44	0.29
6	610	1120	1021	69.3	96.4	58.8	40	48.2	12	14.5	613.1	3510.9	4872	0.2	18.18	0.30
7	770	1190	1037.7	54.1	63.5	57.8	33	42.9	3	3.9	295.1	1885.9	2542.8	0.2	18.08	0.30
8	610	1150	1012	68	96.5	59.3	49	41.9	12	10.3	710.2	3023.7	4204.9	0.2	17.23	0.29
9	500	1130	972.7	98.3	124	61.7	98	50.3	30	15.4	1009.6	5272.6	6409.6	0.2	16.93	0.28
10	600	1130	1000.4	75.9	116.6	60	54	51.9	14	13.5	1214.3	6989.6	8319	0.2	17.98	0.30
11	660	1120	1003.8	51.7	79.2	59.8	28	41	6	8.8	496.5	3707.7	4225.4	0.1	17.55	0.29
12	630	1120	995.1	66.8	100	60.3	39	48.8	9	11.2	362.9	3237.1	3673.1	0.1	18.48	0.31
13	540	1090	986.2	80	119.6	60.8	23	44.2	6	11.5	1326.3	3408.5	4911.6	0.4	17.63	0.29
14	670	1120	1001.3	57.3	82.3	59.9	77	49	16	10.2	403.6	782.4	1703	0.5	16.96	0.28
total (M±SD)	615.00± 84.75	1125.71± 23.21	1004.03± 18.46	71.71± 17.15	98.96± 23.70	59.79± 1.09	46.64± 20.09	46.62± 4.13	12.00± 6.80	11.73± 3.80	725.18± 398.44	3095.64± 1565.28	4108.57± 1828.84	0.26± 0.12	17.31± 0.87	0.29± 0.01
Medium difficulty level																
1	760.00	1080.00	940.80	37.30	58.30	63.80	31.00	36.90	6.00	7.10	263.70	720.00	1059.90	0.40	18.25	0.30
2	740.00	1090.00	946.70	33.10	41.30	63.40	51.00	34.50	7.00	4.70	219.60	451.20	1332.70	0.50	18.28	0.30
3	690.00	1070.00	945.10	47.10	48.40	63.50	52.00	46.00	4.00	3.50	360.90	985.80	1470.80	0.40	19.19	0.32
4	590.00	1100.00	965.10	68.40	96.90	61.50	54.00	35.00	15.00	9.70	1714.02	2448.60	4502.40	0.70	16.51	0.28
5	530.00	1140.00	982.10	54.10	57.80	61.10	88.00	42.90	12.00	5.90	748.10	1159.20	2479.10	0.60	16.29	0.27
6	570.00	1030.00	935.40	68.40	86.80	64.10	73.00	47.70	9.00	5.90	1198.80	1174.90	2953.40	1.00	19.72	0.33
7	490.00	1020.00	951.20	67.70	58.10	63.10	46.00	51.10	6.00	6.70	269.20	206.90	605.90	1.30	17.69	0.29
8	880.00	990.00	927.40	20.20	15.20	64.70	45.00	29.80	0.00	0.00	174.70	192.80	497.70	0.80	18.25	0.30
9	670.00	1050.00	938.50	37.30	51.00	63.90	44.00	46.80	5.00	5.30	174.70	91.10	350.00	1.90	17.39	0.29
10	590.00	1020.00	936.70	59.40	66.90	64.10	59.00	48.80	8.00	6.60	268.80	511.40	1138.40	0.50	19.85	0.33
11	630.00	1090.00	961.70	52.30	67.60	62.40	50.00	50.00	12.00	12.00	193.80	347.00	844.50	0.60	18.02	0.30
12	520.00	1090.00	973.00	69.30	75.00	61.70	50.00	41.30	10.00	8.30	1288.20	3001.70	4613.80	0.40	18.08	0.30
13	860.00	1050.00	984.10	41.30	29.70	61.00	42.00	45.70	6.00	6.50	573.40	284.90	1550.80	2.00	19.20	0.32
14	860.00	1050.00	986.20	40.00	29.30	60.80	40.00	45.50	5.00	5.70	649.30	453.50	1349.10	1.80	17.35	0.29
total (M±SD)	670.00± 127.78	1062.14± 38.21	955.29± 19.20	49.71± 15.01	55.88± 21.74	62.79± 1.29	51.79± 13.71	43.00± 6.31	7.50± 3.72	6.28± 2.68	578.37± 474.54	859.21± 838.59	1767.75± 1328.60	0.92± 0.57	18.15± 1.04	0.30± 0.02
High difficulty level																
1	880.0	1010.0	948.6	26.5	15.4	63.2	56.0	29.5	1.0	0.5	382.3	128.6	650.3	3.0	19.22	0.32
2	590.0	1010.0	809.8	49.6	66.8	74.1	80.0	37.0	24.0	11.1	872.9	785.2	1859.3	1.1	18.73	0.31
3	550.0	1020.0	843.4	45.6	68.9	71.1	88.0	49.2	16.0	8.9	1213.2	983.6	2287.6	1.2	18.43	0.31
4	700.0	1030.0	918.9	47.4	30.6	65.3	110.0	40.9	12.0	5.0	220.4	105.5	804.2	2.1	18.66	0.31
5	780.0	980.0	896.1	44.7	20.5	67.0	87.0	45.5	6.0	3.1	218.9	121.7	525.7	1.8	18.98	0.32
6	210.0	980.0	843.4	87.0	61.0	71.1	183.0	47.3	20.0	5.2	414.5	159.0	1078.1	2.6	18.98	0.32
7	230.0	1980.0	913.8	74.2	121.0	65.7	130.0	44.2	22.0	7.5	203.8	105.9	848.7	1.9	19.32	0.32
8	570.0	1030.0	812.7	50.0	70.1	73.8	86.0	44.6	26.0	13.5	330.2	315.2	776.4	1.1	18.13	0.30
9	720.0	910.0	813.4	36.4	19.4	73.8	95.0	46.3	2.0	1.0	434.4	199.1	909.1	2.2	19.15	0.32
10	660.0	890.0	776.5	33.4	30.3	77.3	70.0	34.8	10.0	5.0	304.9	98.6	1246.0	3.1	18.36	0.31
11	560.0	1040.0	927.7	43.7	55.9	64.7	36.0	28.8	7.0	5.6	554.0	159.8	961.7	3.5	19.11	0.32
12	670.0	1050.0	938.0	37.3	51.0	63.9	44.0	46.8	5.0	5.3	194.7	91.1	550.3	1.9	19.33	0.32
13	660.0	890.0	797.4	44.0	25.8	75.2	85.0	42.1	6.0	3.0	285.7	102.3	528.2	2.8	18.13	0.30
14	630.0	950.0	786.3	36.6	33.3	76.3	111.0	37.0	12.0	4.0	269.4	124.9	896.8	2.2	18.98	0.32
total (M±SD)	600.71± 177.74	1055.00± 261.88	859.00± 59.75	46.89± 15.36	47.86± 27.79	70.18± 4.85	90.07± 35.71	41.00± 6.35	12.07± 7.98	5.62± 3.49	421.38± 279.50	248.61± 268.10	994.46± 490.35	2.18± 0.73	18.82± 0.41	0.31± 0.01

rMSSD – root mean square of successive differences.

Other abbreviations as in Table 1.

The value of the number of breaths per minute with the easy path profile was M±SD 17.3±0.87, a level corresponding to the number of breaths performed under normal conditions.

### Medium level of mission difficulty

The meanRR (Table 2, Figure 2b) from all measurements was M±SD 955.3±49.7 ms. It corresponds to almost 63 bpm. The mean value of the spectral power density for the HF range to the total spectral power of 859.2%, as well as the mean ratio of the spectral power values in the LF range to the spectral power in the HF range of 0.9, indicate a balanced activity of the sympathetic and parasympathetic parts of the nervous system.

The value of the number of breaths per minute with the mean path profile was M±SD 18.1±1.04, a level corresponding to the number of breaths performed under normal conditions.

### High level of mission difficulty

The meanRR (Table 2, Figure 2c) from all measurements was M±SD 859±46.9 ms. It corresponds to >70 bpm. The mean value of the spectral power density for the HF range to the total spectral power of 248.6% and the mean ratio of the spectral power values in the LF range to the spectral power in the HF range of 2.2 indicate increased sympathetic nervous system activity.

The value of the number of breaths per minute with the mean path profile was M±SD 18.8±0.41, a level corresponding to the number of breaths performed under normal conditions.

### Analysis of the results

**Figure 3:**
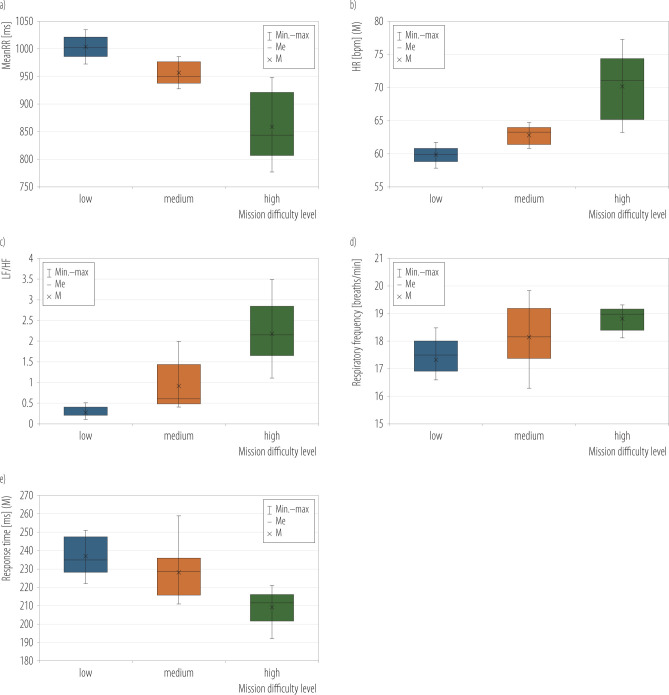
a) The mean RR interval (meanRR), b) heart rate (HR), c) ratio of the spectral power value in the low-frequency (LF) range to the spectral power in the high-frequency (HF) range, d) number of breaths per minute, and e) response time according to mission difficulty level in the research on group of 14 male unmanned aerial vehicles pilots (mean age 27.1 years), Military University of Technology, Warsaw, Poland, April 2025

The meanRR was min. 776.5 ms and max 1037.7 ms at all levels of mission difficulty (Figure 3a). For the low mission difficulty level, a RR of M±SD 1004.03±18.5 ms was obtained. This time reflects approx. 59.8 bpm (SD = 1.1 bpm) (Figure 3b). It is 16.9% more time than the high mission difficulty level (M±SD 859±59.75 ms) and 5.1% more for the medium difficulty level (M±SD 955.29±19.2 ms). The heart rate per minute increased with increasing mission difficulty level (Figure 3b). When performing missions with a medium difficulty level, the mean HR value (meanHR) was 62.8 bpm with SD = 1.3 bpm, an increase of 4.8% relative to missions with a low difficulty level. For missions with a high difficulty level, the increase was 17.4% relative to the easy mission level (M±SD 70.2±4.8 bpm).

The LF/HF ratio reached values 0.1–3.5. The values for the different difficulty levels were M±SD 0.26±0.12 for low, a 3.5-fold increase in the ratio for medium (M±SD 0.91±0.57), and an 8.4-fold increase for high mission difficulty (M±SD 2.18±0.73) (Figure 3c).

In the study presented here, the respiratory rate per minute ranged 14.8–19.85. The values for the different levels of difficulty were M±SD 17.3±0.87 for low, 18.1±1.04 for medium, and 18.8±0.41 for high mission difficulty (Figure 3d).

### Average response time

The subjects' visual-motor RT results ranged 192–259 ms. The RT for the low difficulty level was M±SD 237±9.9 ms. It is 10% slower than for the medium difficulty level (M±SD 228.1±12.3 ms) and 15% for the high difficulty level (M±SD 209±8.1 ms) (Figure 3e).

## DISCUSSION

The heart rate increases as the mission difficulty level increases, evident in the meanRR and meanHR parameters. MeanRR decreases from 1004.03 ms at a low difficulty level of the route to 955.29 ms at a high difficulty level. It is equivalent to an increased bpm as the mission's difficulty level increased from an initial 59.8 bpm through 62.8 bpm at a medium difficulty level to 70.2 bpm for the high difficulty level of the mission.

Heart rate variability analyzed from changes in standard deviation of the RR interval (SDRR) and root mean square of successive differences (rMSSD) parameters suggests sympathetic nervous system activation as the mission difficulty level increases. At a low level of difficulty, a higher mean value of the SDRR and rMSSD parameter is noticeable (SDRR = 71.71 ms, rMSSD = 98.96 ms), suggesting good body adaptation under low-stress conditions. These parameters decrease with increasing mission difficulty and reach SDRR = 49.71 ms and rMSSD = 55.87 ms for medium and SDRR = 46.88 ms and rMSSD = 47.85 ms for high difficulty, respectively. A decrease in the parameters' values may indicate increased stress on the body or the presence of stressful stimuli.

An additional parameter analyzed during the test, which also indicates the activation of the sympathetic part of the nervous system, is the LF/HF ratio. In low-level missions, its low value is noticeable, which suggests a dominance of the parasympathetic nervous system operation. The achieved mean values of LF/HF ratio equal 0.26 are typical of relaxation conditions. An increase in the LF/HF ratio is correlated with an increase in the mission's difficulty level, reaching a maximum M = 2.18.

As the strain on the human body increases due to the increased difficulty level of the mission, an increase in respiratory rate is predicted. The results show that an acceleration of the respiratory rate can be observed, but it is insignificant. It may occur since all subjects were young and had a high level of physical activity. Therefore, the work of their respiratory system may be more efficient and not affected by the tests performed.

Reaction time tests were conducted just after the subjects performed the missions. The average results showed a significantly faster reaction when the test was performed after a high-difficulty mission. The difference is 15% faster for easy missions and 10% for medium-difficulty missions. Such results may indicate the persistence of arousal of the sympathetic part of the nervous system after a completed test using the simulator. It happens because the body is exposed to stress for more complex missions and needs to maintain a higher focus.

As indicated in the relevant literature [30], effective learning is impeded under stress when the response to our actions is immediate, as is the case in the context of UAVs piloting. Furthermore, Porcelli and Degado [31] posit that “chronic stress may support a shift to habitual responding while promoting an insensitivity to novel goal-directed contingencies.” In the context of aviation training, the primary objective is to optimize efficiency by ensuring that pilots are able to assimilate new flight activities with maximum ease and proficiency. Consequently, it becomes imperative to allocate a significant degree of attention to the pilot's stress levels during the training process. It has been demonstrated that it is feasible to establish a mission level that is presently low, high, or of moderate difficulty for the test subject. The automatic selection of mission difficulty levels commensurate with the activation levels of the pilot's autonomic nervous system has been posited as a means of enhancing the efficacy of simulator training.

## CONCLUSIONS

In this study, the main objective was to prove the correlation between the impact of an unmanned aircraft flight simulator and the pilot's body. It has been proven through tests using ECG, PZT, and reaction time measurements that the flight simulator directly induces stress stimuli that affect the subject's body. By analyzing the individual results, it has also been proven that the sympathetic part of the nervous system is activated as the level of difficulty of the missions performed increases.

In future studies, the tests related to reaction time can be extended by performing them several times in quick succession after the mission. It allows us to understand how long the state of arousal of the sympathetic part of the nervous system persists.

Further research into the autonomic nervous system response to stimuli from a UAVs flight simulator is needed. Expanding the current study to include a more extensive study group, including people of more diverse ages, genders, and levels of experience with UAVs flight, as well as testing how much of an impact flying with FPV goggles would have, would enable a more comprehensive assessment of the sympathetic as well as parasympathetic parts of the nervous system.

The data collected and the confirmation of the impact of the UAVs flight simulation allow us to present the hypothesis that it is possible to use the data from the biosensors as mission difficulty controllers, which would result in the pilot, with each simulator exercise being trained in conditions with an optimal level of mission difficulty, adapted to his/her current skills and capabilities. Autonomous selection of the difficulty level would be feasible with artificial neural networks overseeing the training processes.
